# Glycol-Chitosan-Based Technetium-99m-Loaded Multifunctional Nanomicelles: Synthesis, Evaluation, and In Vivo Biodistribution

**DOI:** 10.3390/nano12132198

**Published:** 2022-06-27

**Authors:** Nashmia Zia, Zafar Iqbal, Abida Raza, Aadarash Zia, Rabia Shafique, Saiqa Andleeb, Gilbert C. Walker

**Affiliations:** 1 Department of Pharmacy, University of Peshawar, Peshawar 25120, Pakistan; nashmiazia@gmail.com (N.Z.); zafar_iqbal@uop.edu.pk (Z.I.); 2 Department of Chemistry, University of Toronto, Toronto, ON M5S 3H6, Canada; 3 NILOP Nanomedicine Research Laboratories, Pakistan Institute of Engineering and Applied Sciences, National Institute of Lasers and Optronics College, Islamabad 45650, Pakistan; 4 ARC CoE Convergent Bio-Nano Science and Technology, Monash Institute of Pharmaceutical Sciences, Monash University, Parkville, VIC 3052, Australia; aadarashzia@gmail.com; 5 Department of Biotechnology, University of Azad Jammu and Kashmir, Muzaffarabad 13100, Pakistan; rabiashafique009@gmail.com (R.S.); drsaiqa@ajku.edu.pk (S.A.)

**Keywords:** glycol chitosan, radiotracers, technetium-99m radiolabeling, polymeric micelles, biocompatible, noninvasive imaging, kinetic modeling

## Abstract

We hereby propose the use of stable, biocompatible, and uniformly sized polymeric micelles as high-radiotracer-payload carriers at region-of-interest with negligible background activity due to no or low offsite radiolysis. We modified glycol chitosan (GC) polymer with varying levels of palmitoylation (P) and quaternization (Q). Quaternary ammonium palmitoyl glycol chitosan (GCPQ) with a Q:P ratio of 9:35 (Q9P35GC) offers >99% biocompatibility at 10 mg mL^−1^. Q9P35GC micelles exhibit >99% ^99m^Technetium (^99m^Tc) radiolabeling via the stannous chloride reduction method without heat. The ^99m^Tc-Q9P35GC micelles (65 ± 3 nm) exhibit >98% 6 h serum stability at 37 °C and 7 day of radiochemical stability at 25 °C. HepG2 cells show a higher uptake of FITC-Q9P35GC than Q13P15GC and Q20P15GC. The in vivo 24 h organ cumulated activity (MBq h) order follows: liver (234.4) > kidneys (60.95) > GIT (0.73) > spleen (88.84). The liver to organ ratio remains higher than 2.4, rendering a better contrast in the liver. The radiotracer uptake decreases significantly in fibrotic vs. normal liver, whereas a blocking study with excess Q9P35GC significantly decreases the radiotracer uptake in a healthy vs. fibrotic liver. FITC-Q9P35GC shows in vivo hepato-specific uptake. Radiotracer liver uptake profile follows reversible binding kinetics with data fitting to two-tissue compartmental (2T), and graphical Ichise multilinear analysis (MA2) with lower AIC and higher R^2^ values, respectively. The study concludes that ^99m^Tc-Q9P35GC can be a robust radiotracer for noninvasive hepatocyte function assessment and diagnosis of liver fibrosis. Furthermore, its multifunctional properties enable it to be a promising platform for nanotheranostic applications.

## 1. Introduction

Noninvasive image-guided disease monitoring and therapy is one of the most advanced treatment approaches. It involves tagging of nanoprobes, radionuclides, and fluorescent dyes to drug or targeting agents to track their movement and to enhance the anatomical/physiological changes in the region of interest (ROI) for an adequate time compared to the surrounding tissues [1,2]. At present, ~80% of clinical radiodiagnostic scans are performed with single-positron emission computed tomography (SPECT) and ~20% by positron emission tomography (PET) imaging [3]. SPECT imaging is routinely used to access the hepatic functional reserve, and to diagnose multiple liver pathologies of lesions, fibrosis, cirrhosis, etc. Commercially available SPECT scintigraphy agents for hepatic function assessment are limited and include (technetium-99m) ^99m^Tc-sulfur colloids for reticuloendothelial activity visualization, ^99m^Tc-labeled diethylenetriaminepentaacetic acid galactosyl human serum albumin (GSA) for receptor-mediated hepatocyte function assessment, and ^99m^Tc-labeled iminodiacetic acid (^99m^Tc-IDA) derivatives for hepatobiliary function assessment [4,5]. Among these, GSA is available only in Japan for clinical use [4]. Moreover, the available agents are associated with problems of offsite distribution patterns, higher background signals due to wide size distributions and a high percentage of the colloidal radiotracer, and nonuniform labeling yields upon a change in handling conditions. As in the case of the ^99m^Tc-sulfur colloid kit, pH > 5.5 results in a decrease in radiolabeling rate, and the presence of aluminium may lead to the formation of colloidal radiotracer impurities. Similarly, the interaction of syringe components with ^99m^Tc-IDA leads to an increase in free pertechnetate concentration and the formation of sticky compounds. Moreover, a delay in the addition of ^99m^Tc sodium pertechnetate and hydrochloric acid to the ^99m^Tc-sulfur colloid reagent vial before boiling may lead to the acid reduction of pertechnetate and formation of chelating impurities, which results in greater offsite radiation exposure and lower contrast at ROI [6].

Various strategies have been adopted to enhance contrast at ROIs. These include specific antibody conjugation or the use of nanocarriers (liposomes, polymeric micelles, etc.) for high-payload delivery. Biocompatible polymers such as poly(L-lactide)-, polyethyleneimine-, chitosan-, etc., based nanoparticles are being explored as contrast agent carriers to achieve longer circulation times, lower immunogenicity, narrow size distribution, and preferential biodistribution patterns at ROI [7,8,9].

Numerous ^99m^Tc-labelled nanoplatforms are being developed as SPECT-based contrast agents for molecular imaging of different organs [5,8,10]. Zhang et al. have developed an asialoglycoprotein-receptor-targeting polymeric nanoparticle-based SPECT imaging probe for liver fibrosis [11]. Similarly, the group has also developed a vimentin- and desmin-targeting molecular probe for SPECT imaging of liver fibrosis [12]. These studies indicate that various cell surface receptors or molecular pathways can be exploited to achieve preferential/targeted biodistribution in the liver. For example, palmitic acid accumulates in hepatocytes and plays a central role in the synthesis and maintenance of cell membrane phospholipids and adipose triacylglycerols. The liver is the central organ for the metabolism of saturated fatty acids such as palmitic acid [13]. Moreover, various in vitro and in vivo studies have shown palmitic acid can induce increased lipid droplet accumulation in hepatocytes [13,14,15,16,17]. These findings support the hypothesis that a palmitic-acid-based nanocarrier may accumulate in the hepatocytes. 

Glycol chitosan (GC) and its derivatives are extensively studied as carriers due to ease in functionalization that imparts properties of biocompatibility, mucoadhesion, and ease of modification in biodistribution due to the availability of functional groups [18,19,20,21,22]. Chang-Moon Lee et al. have shown that the hydrophobic modification of the chitosan with linoleic acid can be exploited to increase the hepatocyte-specific uptake of chitosan-coated superparamagnetic iron oxide nanocrystals. Quaternary ammonium palmitoyl glycol chitosan (GCPQ) [23] is a biocompatible polymer with optimum properties for nanoformulation due to the presence of hydrophobic and hydrophilic groups. GCPQ can be modified with varying concentrations of palmitic acid to modify its biodistribution pattern. The presence of quaternary ammonium groups can be utilized to increase the pertechnetate-anion-carrying capacity of micelles to deliver larger payloads of ^99m^Tc [24,25] for better visualization of the liver.

We aim to develop multifunctional polymeric micelles by modifying glycol chitosan with varying palmitoyl and quaternary ammonium substitutions. Radiolabeled micelles have been evaluated for their ease of preparation, radiolabeling efficiency, in vitro stability, biocompatibility, surface hydrophobicity, and high-radiotracer-payload delivery at ROIs with minimum background activity due to low offsite radiolysis. FITC labeling has been used to evaluate liver-specific HepG2 cellular uptake. SPECT imaging and kinetic modeling of ^99m^Tc-loaded radiotracer have also been conducted for an in vivo preclinical demonstration of micelle uptake in a healthy and fibrotic liver animal model. To our understanding, this is the first study to report the development of ^99m^Tc radiolabeled amphiphilic nanomoieties for SPECT-based liver imaging. 

## 2. Materials

Glycol chitosan, 82 kDa (>60% acetylation), methyl iodide (>99%), palmitic acid N-hydroxysuccinimide ester (>98%), Amberlite^®^ IRA-96 free base (>95%), 3-(4, 5-dimethyl thiazol-2-yl)-2, 5-diphenyltetrazolium bromide (MTT) (>97%), Triton X-100 (X100) (>97%), pullulan standards, and solvents were purchased from Merck-Sigma Aldrich Co. (St Louis, MO, USA). Dulbecco’s modified Eagle’s medium (DMEM) supplemented with 10% (*v*/*v*) fetal bovine serum (FBS) (Gibco Life Technologies, Waltham, MA, USA) and penicillin/streptomycin (Gibco Life Technologies, Waltham, MA, USA) was used. NaTcO_4_ (>99%, pH 5.0–5.5) was procured from a ^99^Mo/^99m^Tc Pakgen generator at IPD/PINSTECH, Islamabad, Pakistan. Approximately 20 mg of ^99^Mo was absorbed on an alumina column in the form of ^99^MoO_4_^−2^. Then, 1.0 M normal saline (0.9% NaCl) was used to elute pure ^99m^Tc every 24 h. White male New Zealand rabbits, with a median age of 6 ± 1 month (median ± SEM) and a bodyweight of 1.5 ± 0.2 kg (mean ± SEM), were procured from the National Institute of Health, Islamabad, Pakistan. Animals were housed in controlled laboratory conditions of 22 °C ambient temperature, 60% humidity, and a 12 h light and dark cycle and were treated humanely. Food and water were available ad libitum. Animals were acclimatized to the testing room overnight before the experiment.

## 3. Methods

### 3.1. Synthesis of Polymer with Different Hydrophobic and Hydrophilic Levels and FITC Labelling

Polymers were synthesized through covalent coupling of varying concentrations of palmitic acid N-hydroxysuccinimide (PNS) with hydrolyzed glycol chitosan (HGC), followed by methylation of amino groups to generate amphiphilic polymers [23,26]. Briefly, glycol chitosan (2 g) was acid-hydrolyzed in 4 M HCl (150 mL) for 24 h at 50 °C to produce HGC. HGC (500 mg) and sodium bicarbonate (376 mg) were dissolved in 100 mL ethanol:water (24:76). To this mixture, PNS (1.08 g) in absolute ethanol (250 mL) was added dropwise at varying rates to achieve different levels of palmitoylation. Solutions were stirred at 60 rpm for 72 h. The palmitoyl GC (PGC) was recovered after diethyl ether precipitation and was dialyzed (MWCO 3 kDa) against deionized water (3 L) with 6 changes for 24 h, followed by lyophilization. Quaternization was carried out by dispersing PGC (300 mg) in N-methyl-2-pyrrolidone (25 mL) for 12 h. Sodium hydroxide (40 mg), sodium iodide (45 mg), and methyl iodide (1 g) were added to the reaction mixture and stirred at 60 rpm for 0.5 to 8 h at 36 °C under a nitrogen stream in dark to achieve different levels of quaternary ammonium groups. The product was recovered after diethyl ether precipitation, dissolved in methanol, and dialyzed (MWCO 10 kDa) against deionized water (6 L with six changes over 30 h). The resultant product was lyophilized to give a white cotton-like solid, GCPQ.

FITC (3 mg mL^−1^ in N, N-dimethyl formamide) was added slowly to 0.5 mg of freeze-dried GCPQ polymers in 1 mL sodium bicarbonate buffer (0.2 M). The mixture was allowed to react for 4 h at 60 rpm at room temperature, protected from light. FITC-Q9P35GC, FITC-Q13P15GC, and FITC-Q20P15GC were dialyzed (MWCO 3.5 kDa) against deionized water (2 L) with 3 changes over 48 h and lyophilized. The conjugation of FITC’s isothiocyanate group to amino group was confirmed by NMR spectra (AMX 450 MHz spectrometer, Bruker, Watertown, MA, USA). The appearance of the characteristic aromatic peaks from δ 6.3 to δ 7.4 ppm was identified to confirm the conjugation. 

### 3.2. Physicochemical Characterization of GCPQ Polymers

Gel permeation chromatography (GPC) equipped with a refractive index (RI) detector (Waters 2414, Milford, MA, USA) was used to determine the molecular weight of the unmodified GC and modified GCPQ polymers by comparison to pullulan standards (3–20 kDa). Next, 5 mg mL^−1^ solutions of GC, PGC, and GCPQ in acetate: methanol (50:50) were prepared. The samples were run in 0.5 M acetate (pH 4.9) at a flow rate of 1.0 mL min^−1^ at 30 °C using PL-aqua gel-OH mixed-H column. Critical micelle concentrations (CMC) were calculated as described previously [18,23]. Methyl orange was used as a probe with different polymer concentrations. Hydrodynamic size and zeta potential of micelles were determined by dynamic light scattering (DLS) (ZS-90 Malvern, Westborough, MA, USA).

GCPQ structures were confirmed through ^1^H NMR. Polymer solutions in CD_3_OD: D_2_O (1:3) were prepared at a concentration of 10 mg mL^−1^. ^1^H NMR spectra were recorded by using AMX 450 MHz spectrometer (Bruker, Boston, MA, USA). The levels of palmitoylation and methylation were calculated from spectra at δ: 0.86 (3H, –CH_3_–CH_2_–), 1.1–1.25 (24H, –CH_3_(–CH_2_)_12_–), 1.4 (2H, –CH_2_–CH_2_–CO–), 1.95 (3H, –CH_3_–CO–), 3.3 (9H, (–CH_3_)_3_–N^+^), and 3.4–4.0 (9H, –HO–CH_2_–CH_2_–, and H-3, H-4, H-5, H-6 CH –sugar monomer). Quaternary ammonium to palmitoyl ratio (QPR) was determined to assess the relative strength of hydrophobic vs. hydrophilic groups using the following equations:

(1) $$\%\;Palmitoylation=\left(\frac{\frac{Integration\;value\;of\;N-palmitoyl\;methyl\;protons}{3}}{\frac{Integration\;value\;of\;sugar\;protons}{9}}\right)\times 100$$

(2) $$\%\;Quaternization=\left(\frac{\frac{Integration\;value\;of\;trimethyl\;protons}{9}}{\frac{Integration\;value\;of\;sugar\;protons}{9}}\right)\times 100$$

(3) $$QPR=\frac{mole\;\%\;quaternization}{mole\;\%\;palmitoylation}$$

### 3.3. Surface Contact Angle (SCA) 

Thin films of Q9P35GC, Q13P15GC, and Q20P15GC polymers were prepared following the protocol. The freeze-dried polymer, 10 mg. each, was dissolved in methanol (1 mL) and dried as thin films for 48 h at 25 °C followed by 2 h at 40 °C. Images were captured by adding water drops on thin-film using a DSC-W70 digital camera (Sony, Tokyo, Japan). Analysis was performed through Image J software [27,28]. SCA was calculated to determine the influence of palmitoylation level on the surface hydrophobicity of the GCPQ polymers micelles.

### 3.4. Preparation of Blank and FITC-Labeled GCPQ Micelles

All three polymers (5 mg mL^−1^ each, Q9P35GC, Q13P15GC, and Q20P15GC), dispersed in normal saline (0.9% NaCl, pH 7.4), were probe-sonicated for 10 min, at a 1 s pulse “on” and 5 s “off” at 90 Watt, in ice. Ultrasonic processor GEX 600 sonicator (Cole-Parmer, Quebec, Canada) was set at 75% of its maximum output. 

**Figure 1:**
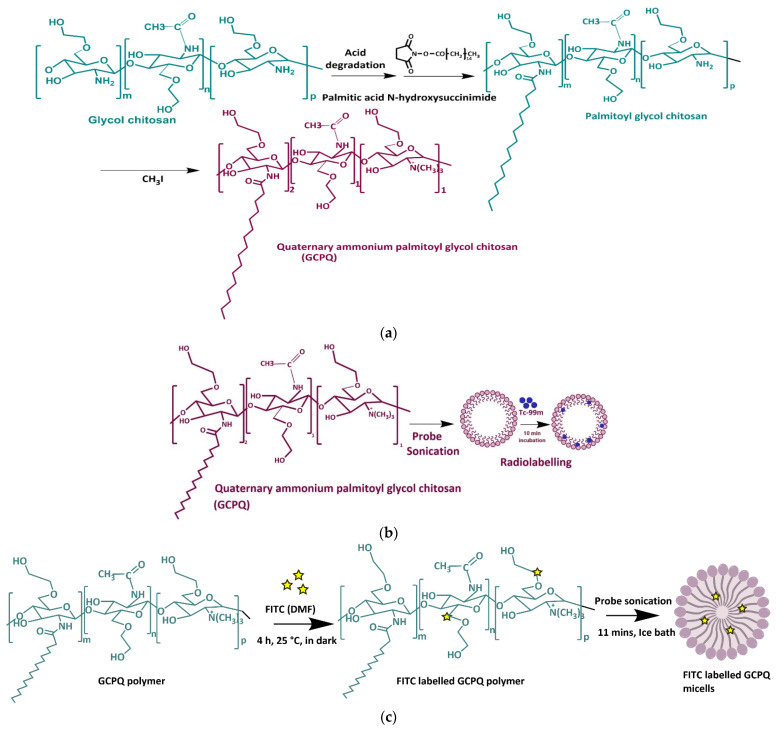
Schematic illustration of glycol chitosan modification, technetium (99mTc) labelling, and fluorescein isothiocyanate (FITC) conjugation. (**a**) Quaternary ammonium palmitoyl glycol chitosan (GCPQ) polymer synthesis scheme; (**b**) GCPQ-99mTc labelling using stannous-chloride-based reduction method; (**c**) GCPQ-FITC labeling; Yellow star represents the FITC molecule.

FITC-GCPQ micelles were prepared following the same experimental conditions used for the preparation of blank micelles (Figure 1). The degree of FITC coupling was determined as follows:

(4) $$\mathrm{Degree}\;\mathrm{of}\;\mathrm{FITC}\;\mathrm{conjugation}=\left(\frac{\frac{\frac{\mathrm{integral}\;\mathrm{at}\;\delta \;6.3-7.4}{\mathrm{number}\;\mathrm{of}\;\mathrm{aryl}\;\mathrm{protons}}}{\mathrm{integral}\;\mathrm{at}\;\delta \;3.4-4.0}}{\mathrm{number}\;\mathrm{of}\;\mathrm{methyl}\;\mathrm{protons}}\right)\times \;\mathrm{DP}\;\mathrm{of}\;\mathrm{GCQP}\;$$

### 3.5. Biocompatibility Determination and HepG2 Cellular Uptake

Biocompatibility was determined using alamar blue assay [29]. Briefly, RAW264.7 cells (20,000/100 µL/well) were seeded in DMEM for 24 h at 37 °C, with 5% CO_2_. Cells were exposed to varying concentrations of GCPQ polymers (1 mg mL^−1^ to 10 mg mL^−1^) in normal saline (0.9% NaCl, pH 7.4), 1% *v/v* Triton-x 100, (positive control) and normal saline (negative control) and left overnight. An amount of 100 µL of alamar blue solution (10% *v/v* in DMEM) was added and cells were incubated for 4 h at 37 °C in a 5% CO_2_ incubator. The fluorescence was measured using FL 6500^TM^ fluorescence spectrofluorometer (Perkin Elmer, Boston, MA, USA) at λ _Ex_ 550 nm, λ _Em_ 590 nm to determine cell viability. All experiments were performed in triplicate. 

To determine in vitro FITC-GCPQ micelles’ liver cell uptake, HepG2 cells in their log phase were seeded in 8-ibidi-well plates (40,000 cells/well), in 200 µL/well starvation DMEM media and incubated overnight at 37 °C in a 5% CO_2_. The starvation medium contained DMEM with 1% penicillin/streptomycin and no FBS. After 24 h, the starvation media was replaced with 100 µL/well of routine DMEM. The cells were subsequently incubated with FITC-Q9P35GC, FITC-Q13P15GC, and FITC-Q20P15GC micelles (5 mg mL^−1^, 100 µL) for 3 h at 37 °C in 5% CO_2_. After incubation, cells were stained with DAPI stain (10 mg mL^−1^) to visualize the nuclei with the confocal laser scanning microscope (Zeiss LSM510, Carlsbad, CA, USA). The images were analyzed using Image J and the difference in the uptake was accessed by applying *t*-tests. 

### 3.6. ^99m^Tc Labeling of Q9P35GC Micelles and Radiolabeling Efficiency

Based on physicochemical properties and HepG2 cellular uptake, Q9P35GC polymer was selected for in vitro and in vivo work. Radiolabelling of Q9P35GC micelles with ^99m^Tc was performed using stannous chloride as a reducing agent under sterile conditions. The radiolabeling efficiency was optimized for a range of SnCl_2_ concentrations_,_ i.e., 100–300 µg at pH 7, by keeping polymer and ^99m^Tc concentrations constant at 5 mg mL^−1^ and 500 MBq, respectively (Table S1). Finally, 50 µL of stannous chloride (2 mg mL^−1^ in 0.1 N HCl) was added dropwise to a 1 mL Q9P35GC micelle dispersion (5 mg mL^−1^ in normal saline, 0.9% NaCl, pH 7.4) in an air-tight vial to prevent opacity. To this mixture, 500 MBq sodium pertechnetate (TcO_4_Na) in 300 µL normal saline was added with moderate shaking at 60 rpm. The final volume was adjusted to 1.5 mL with normal saline. Sodium bicarbonate buffer was used to adjust the pH to 7, and the mixture was allowed to react for 10 min with shaking at 60 rpm to obtain ^99m^Tc-Q9P35GC micelles. The partition coefficient was calculated by distributing ^99m^Tc-Q9P35GC micelles (300 µL, 5 mg mL^−1^) between octanol (500 µL) and PBS (pH 7, 1 X, 500 µL) as organic and aqueous phases, respectively [30]. Radioactivity was determined in both phases using a Genesys Gamma-1 single-well gamma counter (Laboratory Technologies, Inc., Erie, PA, USA). 

The radiolabelling yield was quantified by the double-strip method [31] using instant thin-layer radiochromatography strips (ITLC-SG). ^99m^Tc Q9P35GC micelles and blank (TcO_4_Na) were run on ITLC-SG strips using acetone and normal saline (0.9% NaCl, pH 7.4) as mobile phases separately. Radiochromatograms were analyzed by a gamma-ray spectrometer ITLC Scanner (Bioscan, Washington, DC, USA). 

### 3.7. ^99m^Tc Q9P35GC Micelles’ Size by DLS and TEM, Zeta Potential, Serum Stability, and Hemolysis Study

^99m^Tc-Q9P35GC micelle formulation wet stability was studied for hydrodynamic size and zeta potential for seven days at 25 °C. Serum stability of ^99m^Tc-Q9P35GC micelles was determined at 0.5, 1, 2, 3, 4, and 6 h time points. For serum stability study, the human serum was separated from venous whole blood, collected from a consenting healthy human volunteer, by centrifuging it for 10 min at 1500× *g*, 4 °C. ^99m^Tc Q9P35GC micelle preparation was incubated with normal saline (0.9% NaCl, pH 7.4) and human serum in 1:1 ratio (*v*/*v*) [32] at 37 °C. Radiolabelling stability in serum was checked by determining the percentage of the labeled complex using ITLC-SG as described earlier. To check the effect of plasma protein binding on the physiochemical characteristics of ^99m^Tc Q9P35GC micelles, the hydrodynamic size and zeta potential of the ^99m^Tc Q9P35GC micelles were also determined after incubation with serum. Subsequently, ^99m^Tc Q9P35GC micelle’s hemolytic potential was assessed by following the published protocol [33]. The hydrodynamic sizes of the labelled and blank ^99m^Tc-Q9P35GC micelles were determined by DLS (ZS-90 Malvern, Westborough, MA, USA) followed by transmission electron microscopy (TEM) (Joel 2010, Boston, MA, USA) [34].

### 3.8. In Vivo Biodistribution (SPECT Imaging)

Animals were housed in humane and controlled laboratory conditions of 22 °C ambient temperature, 60% humidity, and a 12 h light and dark cycle. Food and water were available ad libitum. Animals were acclimatized to the testing room overnight before experimentation. Rabbits (*n* = 5) weighing 1–1.5 kg were sedated by intramuscular administration of diazepam (5 mg kg^−1^) and placed in the supine position on a heated bed to maintain body temperature throughout the experiment. ^99m^Tc Q9P35GC micelles (5 mg mL^−1^ in normal saline (0.9% NaCl, pH 7.4), 0.5 mL kg^−1^, 80 ± 2 MBq activity, mean ± SD, *n* = 5) were administered through the marginal ear vein. Immediately after the administration, SPECT dynamic scans were acquired for 25 min using 165 frames (the 20s/projection). Static anterior and posterior scans of 1500 kilo count spot views of the whole body were collected at 0.5, 1, 2, 3, 4, and 24 h with peak energy settings of 140 keV with an energy window width of ±20% using a dual-head gamma GE Infinia Hawkeye GP3 camera (GE Healthcare, LDN, UK). Measurements were corrected for background noise and radioactive decay. The scintigraphic images were processed by ROI analysis using Xeleris Infinia functional imaging workstation software (GE Healthcare, LDN, UK). The biodistribution data were estimated from both static anterior and posterior SPECT scans (0.5, 1, 2, 3, 4, and 24 h) post injection (pi), were normalized as the concentration in the ROI divided by injected dose and multiplied by 100, and were expressed as a percentage of injected dose per organ (%ID).

### 3.9. Organ Cumulated Activity, Absorbed Dose Calculation, and Kinetic Modeling of ^99m^Tc-Q9P35GC Micelles

Cumulated activities in different organs were calculated from the biodistribution data collected at 0.5, 1, 2, 3, 4, and 24 h pi converted to specific activity in Bq and normalized to an injected dose of 80 MBq. These values were plotted vs. time to create a time–activity curve (TAC), and the cumulated activity was computed by integrating this curve using the trapezoidal method and is expressed as MBq h up to the 24 h time point [35]. Based on the MIRD scheme, the total absorbed dose in organs (heart, kidney, bladder, lungs, liver, spleen, and GIT) from administration to 24 h pi was obtained using conjugate (anterior and posterior) counts of organs in static images [36]. 

Liver-to-organ uptake ratios were determined, as an indicator of the liver image resolution, by dividing the activity uptake in the liver by the other three organs’ uptake showing major uptake. TACs of dynamic SPECT scans were generated using Xeleris Infinia functional imaging workstation software for the first 25 min, and standardized uptake values (SUVs) were calculated as a concentration in the ROI divided by injected dose divided by animal weight. Kinetic modeling was performed on the TACs, using an image-derived input function from the left ventricle ROI for each animal scanned by applying General Kinetic Modeling Tool (PKIN) software (version 4.205; PMOD Technologies). Analysis was implemented on a regional basis using compartmental (one-tissue compartmental method (1T model) and two-tissue compartmental model (2T model)) and graphical method of analysis (Ichise Multilinear Analysis (MA2) and Patlak) to estimate tissue concentration (VT) in liver, lungs, and kidneys. 

The selected output for reversible-binding models was tissue concentration (VT), which included the concentrations of all radiotracer in selected tissue (i.e., specific binding and nonspecifically bound), reflecting the equilibrium ratio of radiotracer concentration in the tissue versus blood. VT was expressed in terms of estimated kinetic parameters by VT = k1/k2, for 1-T models and VT = (k1/k2) + (k1 × k3/k2 × k4) for 2-T models. The percentage standard error (% SE) of VT was estimated. A smaller value indicated better identifiability and was used as the selected kinetic model identifiability criterion. The goodness of fit of models was also evaluated using the Akaike information criterion [37] where a lower Akaike information criterion was indicative of a better fit. For graphical analysis, the optimal cutoff time t was determined and the coefficient of determination (R^2^) value was also considered for determining the goodness of fit for these methods.

### 3.10. FITC-Q9P35GC Micelle Distribution in Liver Cells (In Vivo)

Further elucidation of the distribution of Q9P35GC micelles at the cellular level within the liver was conducted by carrying out a FITC/India ink uptake study in healthy New Zealand rabbits. Confocal/optical images were taken to check the distribution of labeled nanoparticles. Rabbits (two groups, *n* = 3) were sedated with intravenous administration of diazepam 5 mg kg^−1^. India ink (1:10 in normal saline 0.9%, 2.5 mL kg^−1^) was injected via the marginal ear vein in one group of rabbits as control. FITC-Q9P35GC micelle (5 mg mL^−1^, 0.5 mL kg^−1^) and India ink were systemically injected into the other group. After 1 h, the rabbits were sacrificed. Tissues were excised and embedded in the optical coherence tomography (OCT) compound before cryostat sectioning. Cryosections (6 µm) were prepared using a cryostat (OTF 5000, Bright, Huntingdon, Cambs, UK). Slides were prepared and H/E-stained. Optical microscopic images were collected. The fluorescence of FITC was visualized using a confocal fluorescence microscope (Zeiss LSM510, Milton, MA, USA) with an excitation filter at 450–490 nm and emission filter of 500–550 nm. Images were recorded using a camera (Neo sCMOS, Andor, UK).

### 3.11. Establishment of Fibrotic Liver Model

To evaluate the effect of fibrosis on the hepatocyte uptake of the Q9P35GC, the liver fibrotic model was established as described previously [12]. Briefly, adult male New Zealand rabbits (*n* = 9) were divided into control (*n* = 3) and test groups (*n* = 6). Test group animals were given 60% carbon tetrachloride (CCl_4_) in olive oil (*v*/*v*) subcutaneously in neck in incremental doses of 0.1 mL kg^−1^ at 1–3 weeks, 0.2 mL kg^−1^ at 4–6 weeks, and 0.3 mL kg^−1^ at 7–9 weeks. The control group animals were given normal saline in the same amount. The development of the fibrosis was confirmed by Masson’s staining of the paraffin-embedded liver tissue.

### 3.12. ^99m^Tc-Q9P35GC SPECT Imaging in Rabbit Liver Fibrotic Model and Blocking Study

To determine the distribution of ^99m^Tc-Q9P35GC micelles in liver tissues of fibrotic rabbits, the rabbits were divided into 2 groups (control, CCl_4_ fibrotic rabbits). Each rabbit was injected with ^99m^Tc Q9P35GC micelles (5 mg mL^−1^ in normal saline (0.9% NaCl, pH 7.4), 0.5 mL kg^−1^, 80 ± 2 MBq activity, mean ± SD, *n* = 5) through the marginal ear vein. Immediately after the administration, SPECT dynamic scans were acquired for 25 min using 165 frames (the 20 s/projection). Blocking experiments were performed with the coinjection of an excess dose of Q9P35GC (20 mg, 0.5 mL kg^−1^), and a static SPECT image was taken after 60 min of administration. The scintigraphic images were processed by the ROI analysis using Xeleris Infinia functional imaging workstation software (GE Healthcare, LDN, UK). The liver distribution was calculated as the concentration in the ROI divided by injected dose and multiplied by 100 and expressed as a percentage of injected (%ID).

### 3.13. Statistical Analysis

Data were analyzed using a one-way analysis of variance (ANOVA) using GraphPad Prism software version 9.1.2 (226) to compare the results of cell viability for varying concentrations of GCPQ polymers and stability of ^99m^Tc-Q9P35GC micelle. An unpaired *t*-test was also used to compare the liver uptake of ^99m^Tc-Q9P35GC micelles in healthy and fibrotic liver rabbits with and without blocking. *p*-values < 0.001 denoted statistically significant differences.

## 4. Results 

### 4.1. Synthesis and Characterization of GCPQ Polymers

In the present study, glycol chitosan was ornamented with palmitoyl and methyl groups that generated Q9P35GC, Q13P15GC, and Q20P15GC corresponding to percent of palmitoylation levels of 35, 15, and 15, respectively, and quaternization levels of 9, 13, and 20, respectively. These were obtained with a change in the rate of PNS feed at 17 mL min^−1^ for Q9P35GC and 11 mL.min-1 for Q13P15GC and Q20P15GC. Methylation reaction duration was 0.5 h for Q9P35GC, 2.5 h for Q13P15GC, and 8 h for Q20P15GC corresponding to QPR ratios of 0.266, 0.867, and 1.33, respectively (Table 1). The level of palmitoylation and quaternization was quantified using ^1^H NMR spectra. The conjugation of the palmitoyl group to the GC was confirmed by the presence of peaks at 0.86 ppm and between 1.1 and 1.25 ppm, which corresponded to the protons on palmitoyl methyl groups. The quaternization of the ammonium group was confirmed by the addition of a peak at 3.3 ppm (Figure S1). The area under the curve was normalized for the respective palmitoyl and quaternary ammonium groups to the sugar protons (Figure S1). The hydrodynamic size was found 75 ± 2 nm for Q9P35GC with the lowest QPR, whereas Q20P15GC, with the highest QPR, showed a size of 125 ± 2 nm, indicating the increase in hydrodynamic size with the increase in QPR. The zeta potential for Q9P35GC, Q13P15GC, and Q20P15GC was 26 ± 1 mV, 24 ± 0.5 mV, and 31 ± 2 mV, respectively. No significant change in zeta potential was observed with the change in QPR but the correlation was found by comparison to pullulan standards. The molecular weights recorded through GPC were 14 ± 1.2 kDa, 12 ± 1.6 kDa, and 14 ± 1.1 kDa for Q9P35GC, Q13P15GC, and Q20P15GC polymers, respectively (Table 1). 

### 4.2. Q9P35GC Exhibited Good Biocompatibility Compared to Q20P15GC, Q13P15GC Polymers against RAW264.7 Cells

**Figure 2:**
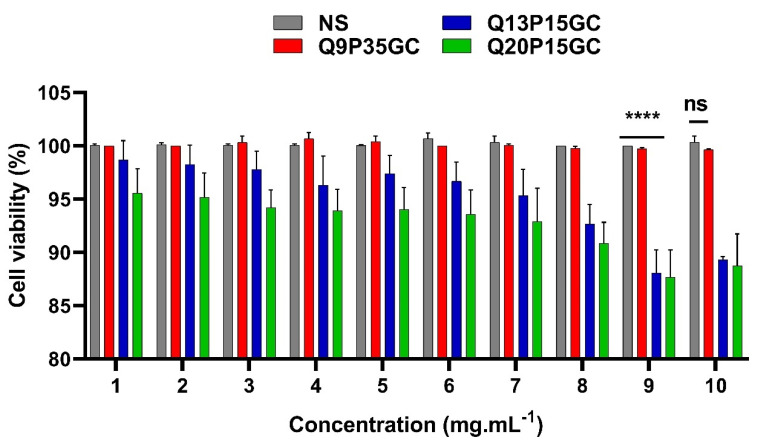
Alamar blue cell viability assay of GCPQ polymers against macrophage (RAW264.7). Q9P35GC showed cell viability equal to normal saline. Unpaired *t*-test. Abbreviations: ns, nonsignificant, NS, normal saline (0.9% NaCl), pH 7.4, ‘****’ shows statistically significant difference with *p* < 0.001.

Cell viability was monitored after incubation of polymer suspensions of Q9P35GC, Q13P15GC, and Q20P15GC in normal saline to examine the concentration-dependent toxicity against RAW264.7 cells. All three polymers showed biocompatibility above 87% at all tested concentrations, i.e., 1 to 10 mg mL^−1^ (Figure 2). Q9P35GC with QPR of 0.257 showed cell viability of >98% at 1 to 10 mg mL^−1^ concentrations which was equivalent to normal saline (control, 0.9% NaCl, pH 7.4). Q13P15GC (QPR: 0.87)- and Q20P15GC (QPR: 1.33)-treated cells showed the viability of >94% at lower concentrations of 1 to 6 mg/mL and the difference in their cell viability compared with normal saline (control, 0.9% NaCl, pH 7.4) was nonsignificant. Cell viability of Q13P15GC and Q20P15GC polymers decreased at higher concentrations of 7 to 10 mg/mL and the difference in the cell viability compared to the normal saline was statistically significant at these concentrations (Figure 2).

### 4.3. SCA Was Higher for Q9P35GC Than for Q20P15GC and Q13P15GC

SCA was used as an indicator of surface hydrophobicity. Increasing the level of palmitoylation from 15 mol % to 35 mol % was found to increase the surface contact angle by 16 ± 5°, indicating a significant and increasing effect on the surface hydrophobicity of polymeric micelles. The SCA was 59 ± 5° for Q9P35GC, whereas it was 43 ± 5° and 41 ± 5° for Q20P15GC and Q13P15GC, respectively. The SCA of Q9P35GC was significantly more than the SCA of the other two polymers, indicating a significant difference in the surface hydrophobicity of the Q9P35GC (Table 1).

### 4.4. Characterization of Blank Q9P35GC and FITC-Q9P35GC Micelles

Q9P35GC and FITC-Q9P35GC micelles showed a mean hydrodynamic diameter of 60 ± 2 nm and 69 ± 5 nm at 25 °C. The count rate was 98.7 (kcps) with a PDI index of 0.361 and 0.32 and the zeta potential was measured to be +28 ± 1 mV and +25 ± 1 mV, respectively. The conjugation of FITC to Q9P35GC was confirmed by the appearance of the characteristic peak of aromatic protons from δ 6.3 to δ 7.4 ppm (Figure S1). The degree of substitution was 12%.

### 4.5. Uptake of FITC-Q9P35GC Micelles in HepG2 Cells Was Higher Than for FITC-Q20P15GC, FITC-Q13P15GC Micelles

**Figure 3:**
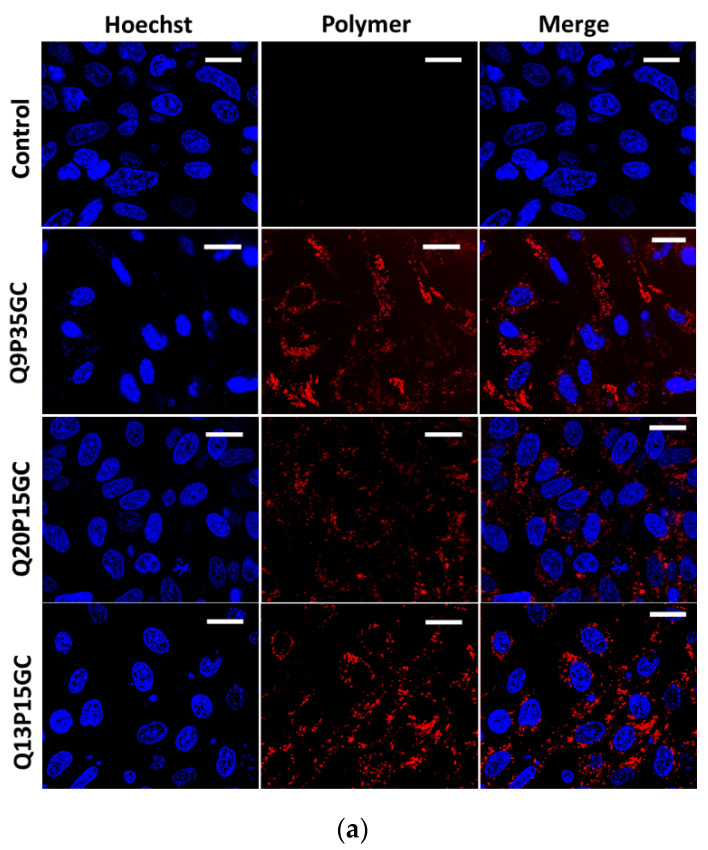

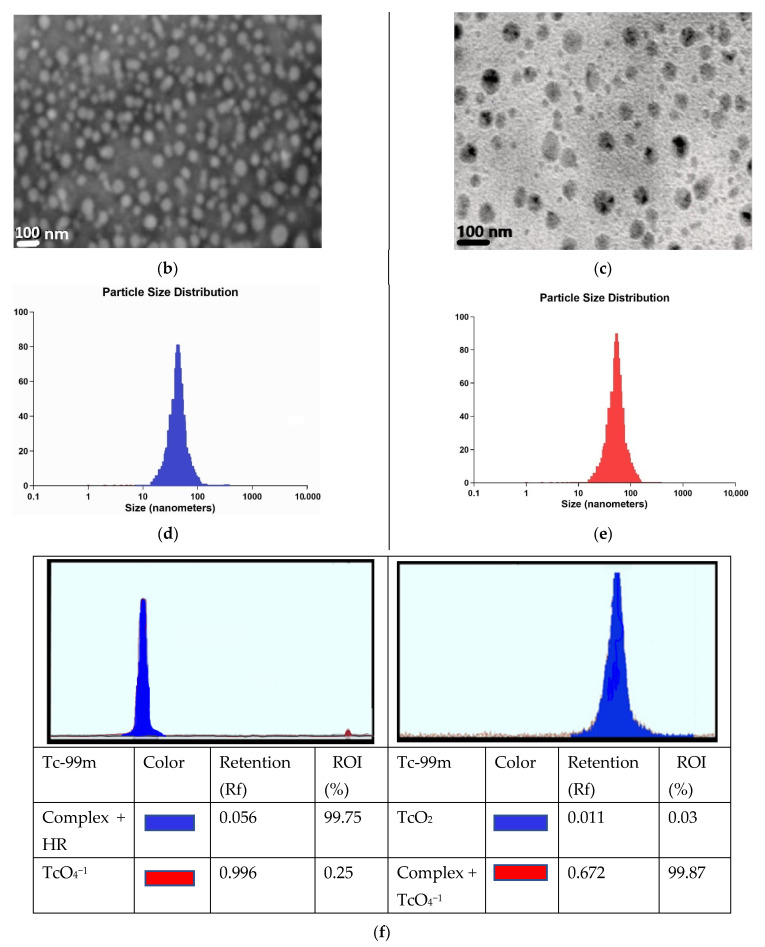
Uptake of FITC-GCPQ micelles (**a**) Live-cell confocal microscopy images of HepG2 cells treated with FITC-Q9P35GC, Q13P15GC, and Q20P15GC micelles. Red fluorescence from FITC GCPQ micelles, the blue flourescence is from nuclei staining, and the last column is the merge. The scale is 20 µm; Characterization of 99mTc Q9P35GC micelles. (**b**) Representative TEM image of Q9P35GC micelles with an average size of 60 ± 9.4 nm, *n* = 6; (**c**) Representative TEM image of 99mTc labelled Q9P35GC micelles with an average size of 65 ± 8.3 nm, *n* = 6; (**d**,**e**) Particle size distribution of Q9P35GC micelles and 99mTc Q9P35GC micelles as determined by DLS, respectively; (**f**) Chromatogram of radiochemical analysis showing labelling efficiency of ^99m^Tc Q9P35GC micelles using double-strip method. Strip one, ITLC-SG Chromatogram of ^99m^Tc Q9P35GC micelles and hydrocolloids at RF = 0 and Free TcO_4_^−1^ at Rf = 1. Strip two, ITLC-SG Chromatogram of hydrocolloids at RF = 0 and Free TcO_4_^−1^ and ^99m^Tc Q9P35GC micelles at Rf = 0.672.

The effect of different levels of palmitoylation on the uptake of the GCPQ micelles in the hepatocyte-specific cell line (HepG2) was evaluated. Quantitative analysis of confocal images by Image J followed by a *t*-test confirmed that FITC-Q9P35GC micelles were taken up by HepG2 cells in significantly higher quantities than FITC-Q20P15GC and FITC-Q13P15GC micelles (Figure 3a). Due to their small hydrodynamic size (65 nm), significantly higher biocompatibility (>99%) at clinically relevant and higher concentrations (10 mg mL^−1^), and increased uptake in HepG2 cells, Q9P35GC was used for radiolabeling and in vivo biodistribution/kinetic studies.

### 4.6. Radiolabeling Efficiency and Physiochemical Characterization of ^99m^Tc-Q9P35GC Micelles

The radiolabeling efficiency of ^99m^Tc-Q9P35GC micelles was more than 99%, as determined by ITLC-SG strips. ITLC’s utility was validated by taking the same mobile and stationary phases for both the sample and blank. Blank-containing pertechnetate showed a different retention factor (Rf) compared to the sample (^99m^Tc Q9P35GC micelle) in both mobile phases. Pertechnetate showed an Rf value of 1 and 0.672 in normal saline and acetone, respectively. ^99m^Tc Q9P35GC micelles and hydrocolloids remained at the origin of the ITLC-SG plate in normal saline (strip 1), giving an Rf value of 0.1 while in acetone (strip 2); hydrocolloids remained at the origin of the ITLC-SG plate giving an Rf value of 0 but ^99m^Tc Q9P35GC micelles moved along with the pertechnetate and gave an Rf value of 0.672 (Figure 3f). The partition coefficient between PBS (1X, pH 7) and octanol showed that ^99m^Tc-Q9P35GC micelles had a log *p* value of 0.3; this value was considered when selecting mobile phases. After labeling, the hydrodynamic size of the ^99m^Tc-Q9P35GC micelles was 65 ± 3 nm at 25 °C with a PDI index of 0.231 (Figure 3d) and zeta potential of +26 ± 1 mV. TEM images were also taken to approximate the size of nanoformulation before and after labeling with ^99m^Tc, as shown in Figure 3b,c.

### 4.7. ^99m^Tc-Q9P35GC Micelles Were Found Biocompatible and Stable

The ^99m^Tc-Q9P35GC micelles showed a nonsignificant change in the hydrodynamic size and zeta potential from day 1 to day 7, with *p*-values of 0.36 and 0.84, respectively, as determined by one-way ANOVA, indicating that the formulation was stable for up to seven days at room temperature (Figure S2a,b). The ^99m^Tc-Q9P35GC micelles were stable up to 6 h with a labeling efficiency of >98% at room temperature in normal saline and in human serum (Figure S2c). The paired *t*-test showed no significant difference in the percentage radiolabeling of the formulation incubated with normal saline and human serum, showing that ^99m^Tc remained bound to the Q9P35GC micelle during the entire study period. The zeta potential of the ^99m^Tc-Q9P35GC micelles decreased after incubation with the serum from +26 ± 1 mV to +16 ± 1 mV, and the hydrodynamic size increased from 65 ± 3 nm to 74 ± 3 nm. The hemolysis was found to be 2.9%, 2.3%, and 0.7% for 0.5 mM (7 mg mL^−1^), 0.05 mM (0.7 mg mL^−1^), and 0.005 mM (0.007 mg mL^−1 99m^Tc Q9P35GC micelles, respectively.

### 4.8. Organ Dose and Cumulated Activity Findings Support Biodistribution Studies

**Figure 4:**
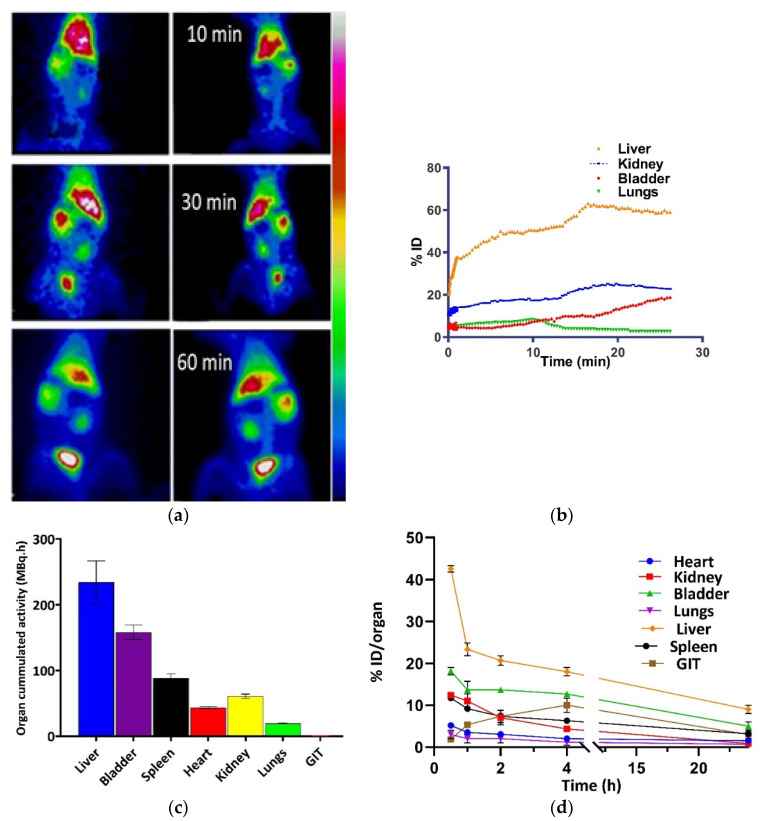
Biodistribution and kinetics of 99mTc Q9P35GC micelles in rabbits. (**a**) Representative SPECT images were taken by Dual-Head Gamma Camera (Anterior: right and Posterior: left) after IV administration of 99mTc Q9P35GC micelles at 10, 30, and 60 min; (**b**) Average SUV TACs in liver, kidney, and bladder during initial 25 min after IV administration of 80 ± 2 MBq of 99mTc Q9P35GC micelles, represented as mean ± SD, *n* = 5; (**c**) Organ cumulated activity following intravenous bolus injection of 99mTc Q9P35GC micelles (80 ± 2 MBq) expressed as MBq h of tissue in different organs (*n* = 3); (**d**) In vivo biodistribution following an intravenous bolus injection of 99mTc Q9P35GC micelles expressed as percentage injected dose with SEM in different organs at different time points. Error bars show SD.

^99m^Tc Q9P35GC micelles’ dynamic SPECT scans showed significant and rapid uptake in the liver, lungs, and spleen followed by the appearance of tracer in the kidney and urinary bladder (Figure 4a). ^9m^Tc Q9P35GC micelles were taken up by the liver within the first few minutes of injection, and the liver time–activity curve reached 95% of its peak activity in approximately 15 min. The images showed good morphological resolution of the liver, as shown in Figure 4a. The percentage distribution of ^99m^Tc Q9P35GC micelles was highest in the liver as opposed to the kidneys, bladder, and lungs, as shown by the first 25 min of their regional time curves (Figure 4b). Around 44 ± 0.8% ID of ^99m^Tc Q9P35GC micelles were found in the liver after 15 min as compared to the kidney, bladder, and lungs having 20 ± 0.9%, 10 ± 0.8%, and 3.8 ± 0.3% ID, respectively. Over time, radiotracer levels decreased in the lungs and spleen, showing only 2.7 ± 0.2% and 11.7 ± 1% ID at 30 min pi, respectively (Figure 4d). ^99m^Tc Q9P35GC micelle cumulated activity in 24 h (Figure 4c) was 234 ± 32.4 MBq h (liver), 60 ± 3.2 MBq h (kidney), 158 ± 10.9 MBq h (bladder), 19 ± 0.7 MBq h (lungs), 88 ± 6.5 MBq h (spleen), and 0.73 ± 0.05 MBq h GIT (Table 2). Results showed a significantly higher accumulation of ^99m^Tc Q9P35GC micelles in the liver compared to other organs. High liver uptake and retention are not the sole determinants of acceptable resolution of liver SPECT images. Liver to other organ ratios such as liver/blood, liver/kidney, and liver/lung are also important (Table S2). The liver to kidney uptake ratios of ^99m^Tc Q9P35GC micelle were 2.85 and 2.7 at 5 and 25 min pi, respectively. The ratio remained greater than 2.4 throughout the study indicating that ^99m^Tc Q9P35GC micelles accumulated much more significantly in the liver compared to other organs. Similarly, the liver-to-blood and liver-to-lungs ratios remained more than 8.1 and 7.1 throughout the study and provided enhanced liver/lung and liver/kidney contrast.

Both renal and hepatobiliary routes of excretion were followed by the radiotracer. Renal excretion appeared to be the dominant removal pathway as indicated by the appearance of radioactivity in the bladder within 30 min of injection of the radiotracer. We observed 18 ± 2.3% ID at 30 pi which increased gradually with time in the bladder with a cumulated activity of 158 ± 10.9 MBq h in 24 h. A smaller percentage of hepatobiliary excretion was also observed, indicated by the appearance of radioactivity in the gastrointestinal tract (GIT). At a time of 30 min after radiotracer administration, 1.9 ± 0.3% of ID was found in different segments of GIT. The activity in GIT decreased over time, and a cumulated activity of only 0.73 ± 0.05 MBq h of radiotracer was seen in GIT (large intestine) in 24 h (Table 2). 

In agreement with the whole-body distribution data (Figure 4), the calculation of the absorbed dose (rad/MBq) in rabbit organs over time (0–24 h), following intravenous bolus administration of ^99m^Tc Q9P35GC micelles (80 ± 2 MBq), also showed a similar pattern of exposure with maximum exposure to the liver and least exposure to GIT at 3.2 ± 0.07 and 0.009 ± 0.0002 rad/MBq ± SD, respectively (Table S3). The percentage clearance of ^99m^Tc Q9P35GC micelles from the injection site was 10% and 18% after 4 h and 24 h, respectively. Fast blood clearance and excretion from the liver and kidney were observed.

### 4.9. Kinetic Modeling

Kinetic modeling was carried out to determine the best-fit method for the quantification of tissue concentration (VT). Results showed that the radiotracer uptake profile in the liver was consistent with reversible binding kinetics, and both modeling methods of analysis (i.e., compartmental and graphical) were found to fit the data. Analysis showed the model preference among animals was for the MA2 reversible graphical analysis model with the regional (liver) AIC of 17.73 ± 4.4 as compared to AICs of 66 ± 0.23 and 69 ± 0.5 for 2T and 1T, respectively. The 2T reversible model was selected as the compartmental model of choice because it provided lower AIC in all datasets (Table 3), especially in regions showing high uptakes, such as the liver and kidney. Similarly, among graphical analysis, MA2 showed higher R^2^ for all regions as compared to Patlak plot analysis, indicating a reversible kinetic model is a better fit to the radiotracer’s kinetics. 

**Figure 5:**
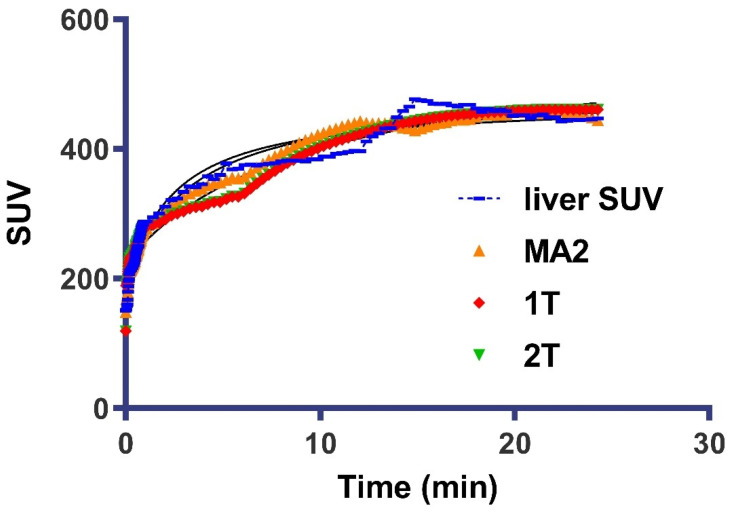
Kinetic modeling of the rabbit liver. Mean liver time–activity curves: Liver standardized value uptake (SUV), blue; red fits by one-compartment model (1T); green fits by two-compartment model (2T); and orange fits by Ichise multilinear regression analysis model (MA2).

Regional VT estimates of liver and fits-to-regional time–activity curves and 1T, 2T, MA2, and Patlak plot are shown in Figure 5. The liver regional VT estimates from MA2 showed the lowest variability in VT (12.42 ± 0.01; coefficient of determination R^2^ = 0.97) and correlate very well with VT estimation by the 2T model (12.66 ± 0.03). The results obtained were compared for each ROI (liver, kidney, and lung), the MA2 model provided a better fit than Patlak graphical analysis, showing a lower AIC (Table 3). The VT ranking order as determined by both models was liver > kidney > lungs. Correlation of 2T and graphical estimation of regional VT (liver) using multilinear regression analysis (MA2) and Patlak plot showed a high correlation of VT estimation during modeling study (25 min) with R^2^ = 0.978 and 0.9312 for MA2 and Patlak, respectively.

### 4.10. FITC-Q9P35GC Micelles Showed Higher Uptake in Hepatocytes in In Vivo Studies

**Figure 6:**
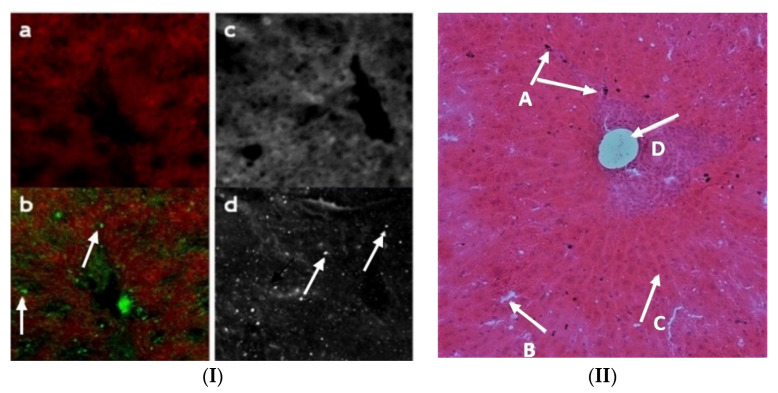
Cellular distribution of ^99m^Tc Q9P35GC micelles. (**I**) Excised liver tissue images captured after 1 h after FITC Q9P35GC/India ink administration; (**a**,**b**) confocal images, green spots of FITC Q9P35GC in hepatocytes and black spot of India ink in Kupffer cells; (**c**,**d**) light microscope images, white spots of FITC Q9P35GC in hepatocytes and black spot of India ink in Kupffer cells, *n* = 3; (**II**) Biodistribution of India ink in the liver 1 h after the administration (as determined by light microscope image at 10×, (**A**) Kupffer cells with India ink, (**B**) sinus spaces, (**C**) hepatocytes arranged in chords, (**D**) portal vein.

The highest cumulative activity was observed in the liver. Post-injection optical images showed uptake of India ink by Kupffer cells which line the liver sinusoid. The cells appeared black because of phagocytosis of carbon particles present in the India ink (Figure 6II). Green fluorescence from the FITC-Q9P35GC micelle was observed in cells other than Kupffer cells. The uptake of India ink by Kupffer cells is an established method for the determination of the phagocytic activity of Kupffer cells [38,39,40,41].

### 4.11. SPECT Imaging and Blocking Studies of ^99m^Tc-Q9P35GC in Fibrotic Rabbit

**Figure 7:**
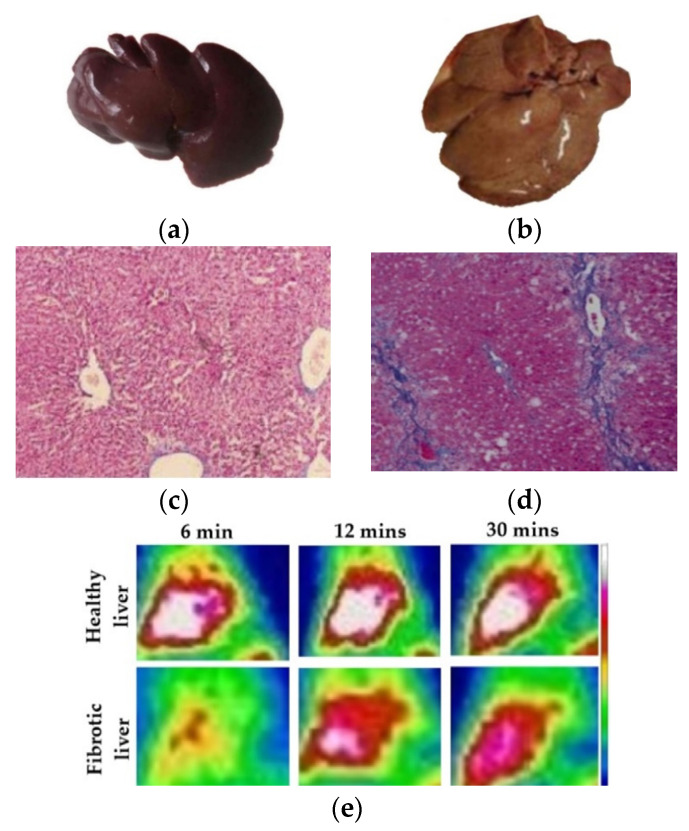
Characterization of a CCl_4_-induced rabbit model of liver fibrosis. Representative images of: (**a**) Normal liver; (**b**) Fibrotic liver: Masson’s stained liver tissue of; (**c**) control; (**d**) fibrotic liver after CCl_4_ administration for 9 weeks, fibrotic liver tissue is showing increased collagen (purple); (**e**) Representative ^99m^Tc-Q9P35GC dynamic SPECT images at different time points for fibrotic and healthy liver.

**Figure 8:**
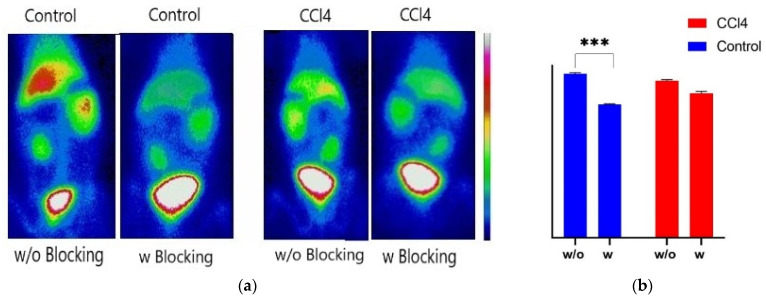
Blocking studies of 99mTc-Q9P35GC in fibrotic mice. (**a**) Representative SPECT image of healthy and fibrotic-liver rabbit at 60 min pi 99mTc-Q9P35GC (80 MBq) with or without Q9P35GC for blocking. Images were adjusted using the same scale for all animals; (**b**) Hepatic uptake of 99mTc-Q9P35GC derived from SPECT imaging by drawing the ROI of the whole liver, uptake is normalized for each group, ‘***’ shows statistically significant difference with *p* < 0.001.

The uptake of ^99m^Tc-Q9P35GC in the rabbits with fibrotic liver was lower as compared to the liver of healthy rabbits (Figure 7). To confirm the hepatocyte specificity of ^99m^Tc-Q9P35GC in the fibrotic liver, blocking experiments were performed. After the coinjection of an excess dose of Q9P35GC (20 mg), the hepatic uptake of ^99m^Tc-Q9P35GC was significantly reduced in healthy rabbits (*p* ˂ 0.001; Figure 8a,b). By contrast, there was a nonsignificant decrease in the hepatic uptake of ^99m^Tc-Q9P35GC in the liver of fibrotic rabbits, indicating that it could be due to a lower number of functional hepatocytes.

## 5. Discussion

The use of biocompatible polymeric nanomicelles for SPECT-based noninvasive imaging offers the advantage of better resolution of organs, high radiolabelling yield, and a high signal-to-noise ratio. However, the chemical composition of the polymer has to be optimized for biocompatibility and organ specificity without compromising the physical properties needed for in vitro labelling efficiency and optimum in vivo biodistribution [42,43,44,45]. Herein, we have described the preparation of three glycol chitosan polymers with different levels of palmitoyl and methyl group substitutions to impart increasing surface hydrophobicity to increase the hepatocyte-specific uptake of the nanoparticles [10,46]. The Q9P35GC polymer with the highest surface hydrophobicity is the optimal variant due to its highest HepG2 cell uptake. This is similar to the findings reported by Lee et al., that surface coating of SPIONs with linoleic acid, a saturated fatty acid, increased the surface hydrophobicity and hepatocyte uptake of the nanoparticles. Moreover, the Q9P35GC polymer also showed 99% biocompatibility at concentrations as high as 10 mg mL^−1^. This is important as high radiotracer concentrations are usually used in clinical administrations to achieve statistically significant ROI resolutions, e.g., a 3–10 mg dose of the ^99m^Tc-labelled glycosylated serum albumin (^99m^Tc GSA) is typically used for liver imaging in human subjects [47].

The Q9P35GC also exhibited ideal properties for micelle synthesis. The low CMC of 0.13 µg.mL^−1^ imparts high-radiolabelled-formulation stability when diluted in blood during in vivo studies and has a higher chance of retaining the imaging agent within the micellar structure. This is a desired feature in SPECT radiotracers as it limits non-site-specific radiolysis [45,48,49]. The ^99m^Tc-Q9P35GC micelle also possesses in vitro radiolabelling stability, as indicated by the minimal formation of colloidal ^99m^Tc (<2%) when incubated with serum for 6 h at room temperature. The stability of the ^99m^Tc-Q9P35GC micelle has also been analyzed by assessing the size and zeta potential for 7 days. No significant changes were observed, indicating a highly stable formulation [43] that can be prepared beforehand in large quantities for convenient use.

Major factors limiting the clinical translation of SPECT-based radiotracers are their time-consuming and complex synthesis processes, low labelling yields, and nonuniform size distributions [45]. However, the work herein utilized a one-pot [50] SnCl_2_ reduction method for the radiolabelling of Q9P35GC with ^99m^Tc to generate a >99%-labelling-yield radiotracer. It is in coherence with the idea suggested by Piarciovà et al. that the use of a reducing agent will lower the ^99m^Tc oxidation state and increase the sorption of the pertechnetate anion to chitosan [24]. Moreover, the radiolabeling of Q9P35GC micelles was carried out at room temperature with 10 min reaction time, without the use of heat. This synthesis procedure is very simple as compared to other published protocols which required high temperatures and a longer duration for radiolabelling of polymeric preparations. Zhang et al. have reported a copolymer-based SPECT-imaging agent preparation which required 24 hr incubation with the gadolinium and 30 min incubation with heating at 60 °C for Tc-99m radiolabeling, respectively [51]. 

^99m^Tc Q9P35GC micelles’ biodistribution data show rapid and maximum accumulation in the liver within 15 min pi; this characteristic is comparable to the already available preparation of ^99m^Tc-GSA [52] and ^99m^Tc-labeled galactosyl methylated chitosan (^99m^Tc-GMC) which showed rapid uptake of 12% and 27% in the liver at 10 min pi, respectively [53]. Moreover, the liver to kidney uptake ratio of ^99m^Tc Q9P35GC micelles (2.9) is also comparable to that of ^99m^Tc GSA (5.7) [52]. The entrapment of ^99m^Tc Q9P35GC micelles primarily in the liver is comparable to a study by Banerjee et al. [54]. However, this distribution pattern is attributed to the tin-colloid formation and its interaction with chitosan nanoparticles to form large particles in the micron range, which is not the case in our study. Although the presence of different impurities such as ^99m^TcO_2_ and tin colloids cannot be ruled out, their presence could not have significantly affected the observed biodistribution pattern due to their low concentrations, i.e., <1% of particles are found to be greater than 120 nm in diameter.

The biodistribution of radiotracers inside the body is observed to depend on many factors, such as size, charge, orientation, etc., which dictate their interaction with plasma proteins [55,56]. We observed the same pattern of altered biodistribution when using ^99m^Tc Q9P35GC micelles as compared to that of free ^99m^Tc, which accumulates mainly in the stomach, gastrointestinal tract, thyroid, salivary glands [57]. ^99m^Tc Q9P35GC micelles are distributed in negligible amounts in these organs, which further supports the in vivo radiostability of the prepared radiotracer. 

Both surface hydrophobicity and surface charge are known to determine the amount and composition of protein in the corona on polymeric and inorganic nanoparticles. The characterization of surface hydrophobicity’s effect on the protein corona composition of amphiphilic polymeric nanoparticles is complex due to the presence of hydrophobic and hydrophilic groups in varying amounts. Bewersdorff et al. have reported a higher concentration of albumin and apolipoprotein on the surface of moderately hydrophobic nanoparticles as compared to the highly hydrophobic [58]. The formation of a protein corona was speculated by comparing the surface charge and hydrodynamic size of the bare and corona-adsorbed ^99m^Tc Q9P35GC micelles. The change in the zeta potential showed the adsorption of negatively charged proteins. It has been observed that moderately hydrophobic and cationic nanoparticles tend to adsorb albumin [59], which is a dysopsonin and helps in endocytosis [60], evasion of macrophage-based cellular uptake by the RES system in the liver, and promotes longer circulation times [38,61,62,63]. We propose that ^99m^Tc Q9P35GC, being moderately hydrophobic and cationic, would also bind preferably to the albumin and that should enhance longer circulation and RES evasion. However, more in-depth proteomic analysis and protein quantification is needed to predict the effect of the protein corona on the surface properties and uptake of Q9P35GC micelles under in vitro and in vivo conditions.

Our study suggests that increasing the palmitoylation at lower molecular weight leads to an increase in the surface hydrophobicity, as shown by significant increase in SCA, which resulted in the higher hepatocyte-specific uptake of ^99m^Tc Q9P35GC micelles. In contrast, Alameh et al. have observed that the biodistribution of chitosan nanoparticles in the liver (RES) increased with Mn [1,64]. The increase in SCA can be attributed to the increase in the palmitoyl pendant chains available on the surface of the GCPQ micelles, as observed by Chiu et al., and that increasing the hydrophobic substitution of chitosan results in the availability of the hydrophobic groups on the surface of micelles, which increases surface hydrophobicity and in turn hepatocyte-specific uptake [10,65].

Renal pathways are observed to be a major route of excretion, as shown by the presence of a large amount of radioactivity in the bladder, which is in coherence with the expected route of excretion for hydrophilic polymeric substances [66]. This observation is like that made by Yang et al., who observed that ASGP-R-targeted co-polymer complex-based SPECT-imaging agent 99mTc[P(VLA-co-VNI)] (tricine) followed renal excretion when not bound to receptors [53,67]. Renal excretion is also observed for other ASGPR-targeted radiotracers including ^99m^Tc-GSA, ^99m^Tc-GMC, and fluorine-18-labeled GSA (^18^F-GSA) [53]. Other studies have also reported the excretion of glycol-chitosan-based nanoparticles via the renal excretion route [68]. 

Multiple studies have reported that the phagocytosis of India ink by Kupffer cells renders them black due to the presence of carbon particles [41,69]. FITC-Q9P35GC micelles also show a bright green spot within the cells other than Kupffer cells. Moreover, cell uptake study of FITC-GCPQ micelles in the HepG2 liver-specific cell line showed that uptake increased with an increase in palmitoylation levels, indicating that accumulation of FITC-Q9P35GC micelles in the liver can be attributed to its uptake by hepatocytes. Other studies have also reported the specific accumulation of chitosan and its derivatives within hepatocytes [70,71]. It was reported previously that the number of functional hepatocytes decreases significantly in the fibrotic liver of rabbits, especially in advanced fibrosis. In this study, we evaluated the hepatospecificity of the ^99m^Tc-Q9P35GC by investigating change in its liver uptake within fibrotic livers. High-quality images of the whole liver were obtained using ^99m^Tc-Q9P35GC by SPECT imaging in a CCl_4_-induced fibrotic rabbit. We demonstrated that SPECT imaging with ^99m^Tc-Q9P35GC could potentially show a decrease in the functional capacity of hepatocytes in liver fibrosis, 9 weeks after CCl_4_ treatment. The pathologic process of fibrosis and a significant decrease in hepatocytes were confirmed by histologic staining. 

All the desired radiotracer characteristics listed by W. Yang et al. for liver imaging, including high radiolabelling efficiency, in vivo stability, high cumulation in the liver, and excretion preferably through the kidneys [53], were shown by our synthesized ^99m^Tc Q9P35GC radiotracer [53]. Kinetic modeling of ^99m^Tc Q9P35GC micelle uptake in the liver showed reversible binding characteristics. Further uptake studies are required to identify the receptors involved in ^99m^Tc Q9P35GC micelle uptake within hepatocytes and its fate inside the liver. 

In conclusion, the developed ^99m^Tc Q9P35GC micelles are promising candidates for noninvasive imaging as they show a high radiochemical stability, radiolabeling efficiency and higher contrast in the liver. The biocompatible ^99m^Tc Q9P35GC micelle presented no agglomeration in serum or normal saline, an important factor for in vivo application of these micelles as it will permit dependable and repeatable imaging. The easy one-pot technitium-99m labeling method and lack of biohazards associated with the use of blood-based products make ^99m^Tc Q9P35GC micelles a good candidate for SPECT imaging. 

## Figures and Tables

**Table 1:** Physiochemical characterization of GCPQ polymers.

QxPyGC Polymer	Molecular Weight of GCPQs (kDa) ^a^	Mole Percent Palmitoyl Group (%) ^b^	Mole Percent Quaternary Ammonium Group (%) ^c^	CMC (gL^−1^/µM) ^d^	QPR	Zeta Potential (mV)	Hydro-Dynamic Size (nm)	PDI	Surface Contact Angle (SCA)
Q9P35GC	14 ± 1.2	35	9	0.13 ± 0.03	0.26	2.6 ± 1	75 ± 2	0.28 ± 0.02	59 ± 5
Q13P15GC	12 ± 1.6	15	13	0.15 ± 0.04	0.86	2.4 ± 0.5	80 ± 1	0.42 ± 0.12	43 ± 5
Q20P15GC	12 ± 1.1	15	20	0.23 ± 0.02	1.33	3.1 ± 2	125 ± 2	0.39 ± 0.07	41 ± 5

The nomenclature of the prepared GCPQ polymers is presented as QyPxGC, where ‘x’ is the mole percent of the quaternary ammonium group substitution and ‘y’ is the mole percent of the palmitoyl group substitution. ^a^; Weight of GCPQ after chemical modifications using gel permeation chromatography, ^b^; Calculated using the ratio of palmitoyl methyl protons to sugar protons in palmitoylated PGC, using NMR, ^c^; Calculated using the ratio of quaternary ammonium to sugar protons in GCPQ, using an NMR, ^d^; Critical micelle concentration, determined by a fluorescence-quenching method.

**Table 2:** Cumulated activity in rabbit organs over time (0–24 h), following intravenous bolus administration of 99mTc Q9P35GC micelles (80 ± 2 MBq). Values in MBq h, presented as mean ± SEM (*n* = 5).

	0–0.5 h	0.5–1 h	1–2 h	2–4 h	4–24 h	0–24 h
Heart	1.4 ± 0.06	2.7 ± 0.10	4.5 ± 0.2	5.9 ± 0.2	29 ± 1.1	43 ± 1.7
Kidney	2.3 ± 0.1	4.4 ± 0.2	6.8 ± 0.4	8.5 ± 0.4	39 ± 2	60 ± 3.2
Bladder	3.3 ± 0.1	5.7 ± 0.2	9.7 ± 0.7	19 ± 1.3	125 ± 8.6	158 ± 10.9
Lungs	0.6 ± 0.02	0.9 ± 0.03	1.5 ± 0.05	2.4 ± 0.08	14 ± 0.5	19 ± 0.7
Liver	6.8 ± 0.9	12.2 ± 1.7	17.3 ± 2.4	25 ± 3.4	173 ± 23.9	234 ±. 32.4
Spleen	2.1 ± 0.1	3.7 ± 0.3	5.9 ± 0.4	9.7 ± 0.7	67 ± 4.9	88 ± 6.5
GIT	0.1 ± 0.01	0.02 ± 0.00	0.03 ± 0.00	0.04 ± 0.00	0.54 ± 0.04	0.73 ± 0.05

**Table 3:** ^99^mTc Q9P35GC micelles’ kinetic-modeling data.

	2 T Model			1 T Model			Multilinear Analysis (MA2)		
	VT (ml/ccm) (Mean ± SEM)	% SE (Mean ± SEM)	AIC (Mean ± SEM)	VT (mL/ccm) (Mean ± SEM)	% SE (Mean ± SEM)	AIC (Mean ± SEM)	VT (mL/ccm) (Mean ± SEM)	AIC (Mean ± SEM)	R^2^
Liver	12.66 ± 0.03	1.4 ± 0.1	66 ± 0.23	12.1 ± 0.05	16.4 ± 0.28	69 ± 0.5	12.42 ± 0.01	17 ± 4.4	0.97
Lungs	1.08 ± 0.06	2.8 ± 0.1	382.6 ± 0.54	1.09 ± 0.09	4.99 ± 0.03	386 ± 12.2	5.07 ± 0.6	209 ± 3.9	0.86
Kidney	8.3 ± 0.1	1 ± 0.2	39.3 ± 0.2	8.9 ± 0.1	1.9 ± 0.01	7.1 ± 0.17	21.5 ± 2.05	12 ± 0.5	0.664

Volume of tissue concentration (VT) estimation through 1T, and 2T compartmental model and MA2 graphical analysis using standardized uptake value (SUV) data to derive the input function (*n* = 5); % Standard error (% SE); Akaike information criterion (AIC).

## Data Availability

Not applicable.

## Supplementary Materials

The following supporting information can be downloaded at: https://www.mdpi.com/article/10.3390/nano12132198/s1, Figure S1: ^1^H NMR of different GCPQ polymers and FITC Q9P35GC polymer; Figure S2: In vitro stability of ^99m^ Tc Q9P35GC micelles; Table S1: Optimization of radiolabeling efficiency of ^99m^Tc Q9P35GC micelles by varying the concentration of reducing agent; Table S2: Liver to non-target tissue ratios in rabbits based on the radiotracer biodistribution calculated as % ID ± S.D; Table S3: Absorbed dose in rabbit organs over time (0–24 h), following intravenous bolus administration of ^99m^Tc Q9P35GC micelles.
