# Supplementary material for: Glycol-Chitosan-Based Technetium-99m-Loaded Multifunctional Nanomicelles: Synthesis, Evaluation, and In Vivo Biodistribution
Source: Nanomaterials (Basel). 2022 Jun 27;12(13):2198. doi: 10.3390/nano12132198 (PMC9268087; doi:10.3390/nano12132198)
Supplement: Supplementary file 1 [file nanomaterials-12-02198-s001.zip › nanomaterials-1736811-supplementary.pdf]

Supplementary Information

Glycol Chitosan Based Technetium-99m Loaded Multifunctional Nanomicelles: Synthesis, Evaluation and In Vivo Biodistribution

Nashmia Zia [1,2], Zafar Iqbal [1], Abida Raza [3,*], Aadarash Zia [4], Rabia Shafique [5], Saiqa Andleeb [5] and Gilbert C. Walker [2,*]

1 Department of Pharmacy, University of Peshawar, Peshawar 25120, Pakistan; nashmiazia@gmail.com (N.Z.); zafar_iqbal@uop.edu.pk (Z.I.);

2 Department of Chemistry, University of Toronto, Toronto, ON, M5S 3H6, Canada;

3 NILOP Nanomedicine Research Laboratories, Pakistan Institute of Engineering and Applied Sciences, National Institute of Lasers and Optronics College, Islamabad 45650, Pakistan;

4 Monash Institute of Pharmaceutical Sciences, Melbourne, Australia; aadarashzia@gmail.com (A.Z.);

5 Department of Biotechnology, University of Azad Jammu and Kashmir, Muzaffarabad 13100, Pakistan; rabiashafique009@gmail.com (R.S.); drsaiqa@ajku.edu.pk (S.A.)

* Correspondence: abida_rao@yahoo.com (A.R.); gilbert.walker@utoronto.ca (G.C.W.); Tel.: + 92-345-7713910 (A.R.); + 1-416-427-4912 (G.C.W.)

Results:

(a)

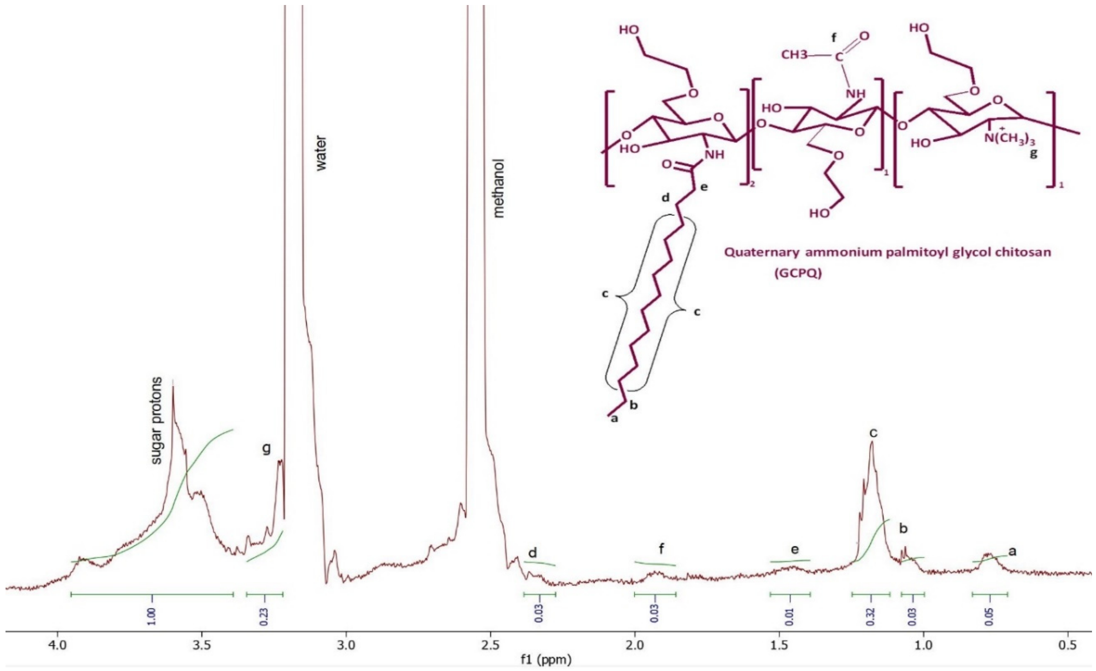

water
methanol
sugar protons
g
d
f
e
c
b
a
1.00
0.23
0.03
0.03
0.01
0.32
0.03
0.05
4.0
3.5
3.0
2.5
2.0
1.5
1.0
0.5
f1 (ppm)
Quaternary ammonium palmitoyl glycol chitosan
(GCPQ)

(b)

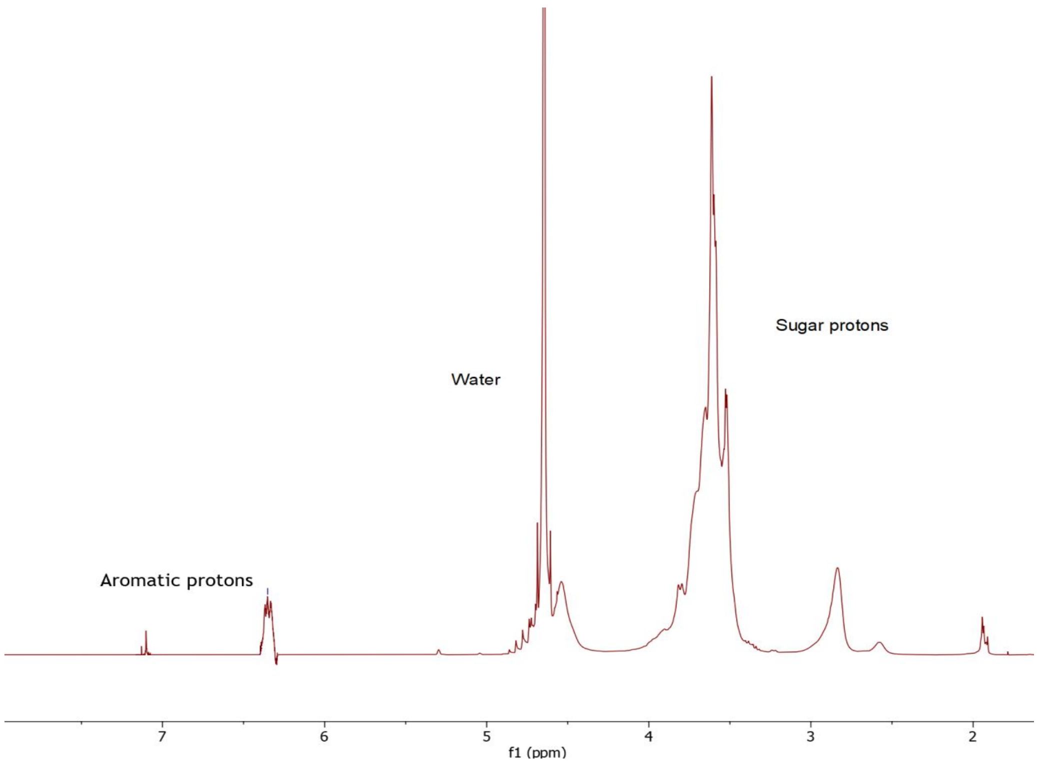

Aromatic protons
Water
Sugar protons
7
6
5
4
3
2
f1 (ppm)

(c)

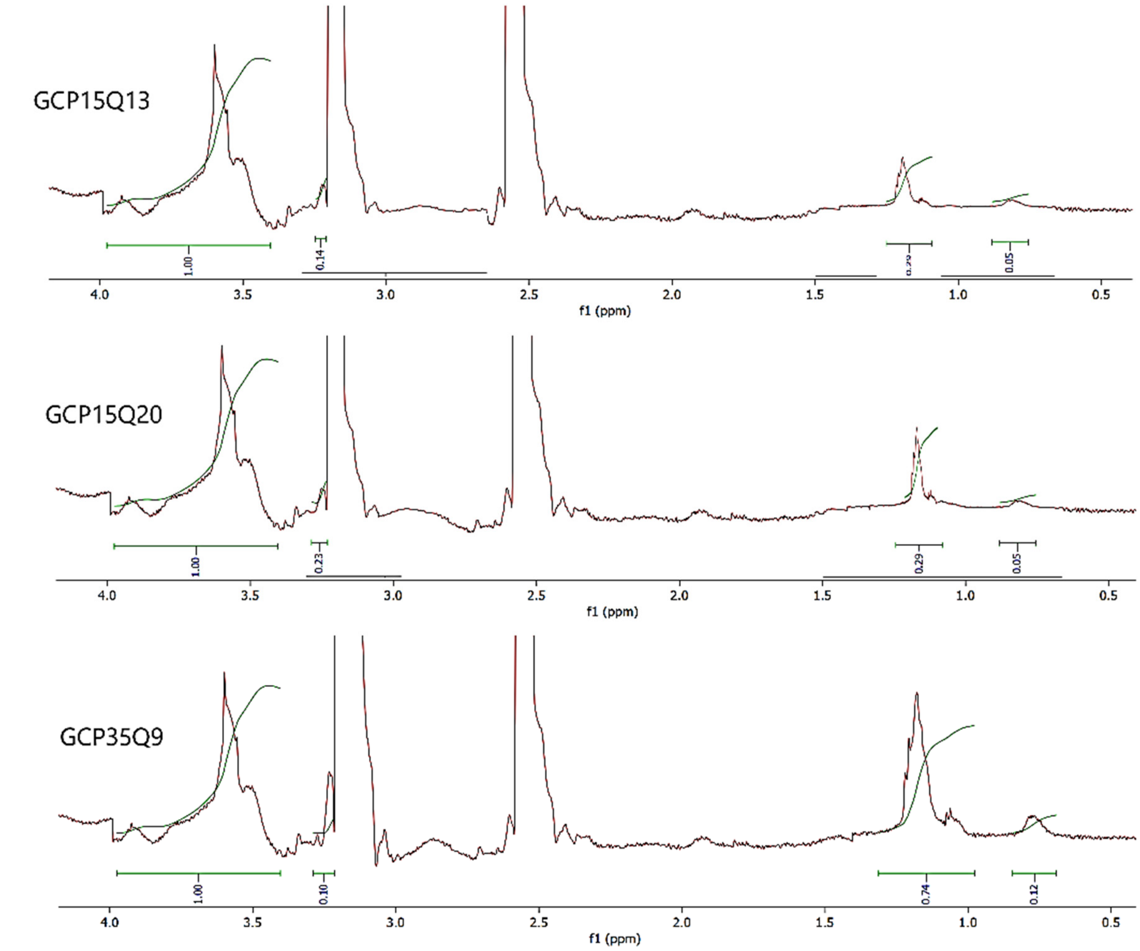

Figure S1. ^{1}H NMR of different GCPQ polymers and FITC Q9P35GC polymer; as measured by AMX 450 MHz spectrometer (Bruker, Watertown, MA, USA), 10 mg.mL^{-1}, D_2O:CH_3OD (3:1) **a)** ^{1}H NMR spectra of GCPQ polymer, ^{1}H-NMR (CD3OD, D_2O, 1.0:3.0) δ: 0.86 (3H,–*CH_3* CH_2), 1.1-1.25 (24H,CH_3(*CH_2)$_{12}$*), 1.4 (2H,*CH_2* CH_2 CO), 1.95 (3H,*CH_3* CO), 3.3 (9H, (*CH_3)$_3$* N^+), 3.4 - 4.0 (9H, HO*CH_2* *CH_2* and H-3, H-4, H-5, H-6 CH –sugar monomer); **b)** ^{1}H NMR spectra of FITC Q9P35GC, ^{1}H-NMR (D_2O, CH_3OD, 3.0:1.0): 1.95 (3H, CH_3CO), 3.3 (9H, $(CH_3)_3N+$), 3.4 - 4.0 (9H, $HOCH_2CH_2$ and H-3, H-4, H-5, H-6 CH –sugar monomer), 6.3 – 7.4 (9H, (-CN- aryl protons) **c)** Stacked ^{1}H NMR of Q9P35GC, Q20P15GC, and Q20P15GC. NMR peak spectrum is integrated for sugar backbone (1.0), palmitoyl group (0.05, 0.05, and 0.12) and quaternary ammonium group (0.20, 0.13, and 0.1) for Q20P15GC, Q13P15GC, and Q9P35GC, respectively.

(a) **(b)**

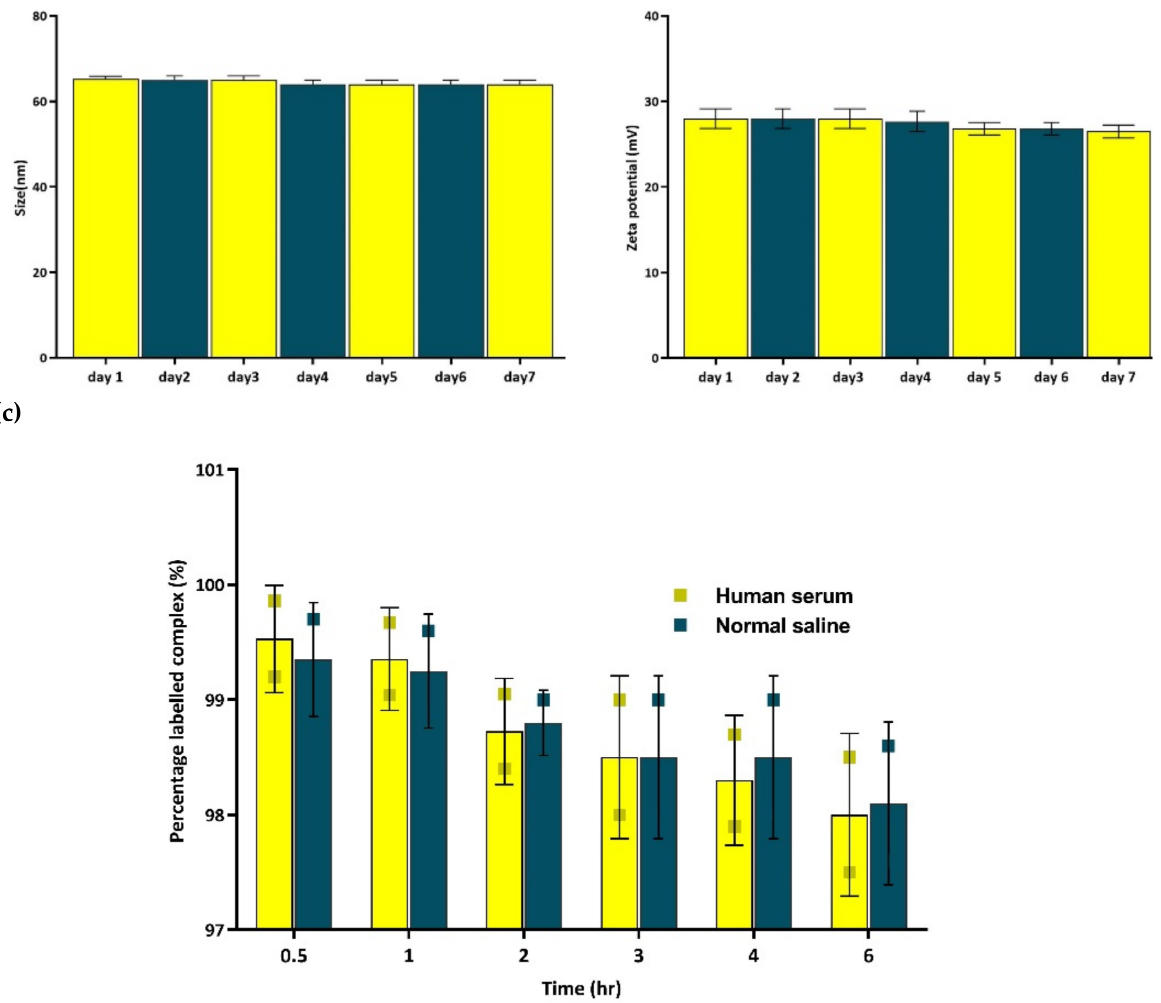

Figure S2. *In vitro* stability of [99m] Tc Q9P35GC micelles. **a)** *In vitro* size stability of [99m]Tc Q9P35GC micelles over a period of 7 days, presented as mean with SD, n=3 at room temperature, No significant difference in the size was observed as determined by one-way ANOVA, p=0.36; **b)** *In vitro* zeta-potential stability of [99m]Tc Q9P35GC micelles over 7 days presented as mean with SD, n=3 at room temperature, No significant difference in the zeta potential was observed as determined by one-way ANOVA, p=0.84; **c)** In vitro radiolabeling stability of [99m] Tc Q9P35GC micelles at 37 °C in normal saline and human serum over a period of 6 h, with the standard error of the mean (SEM), n=3. Paired t-test.

Table S1. Optimization of radiolabeling efficiency of ^{99m}Tc Q9P35GC micelles by varying the concentration of reducing agent.

$SnCl_2$ (μg)	Q9P35GC micelles (mg)	99m Tc Activity (± 20 MBq)	Temperature (°C)	% age radiolabeling
300	5	500	25	Free 5%, labeled 95%
200	5	500	25	Free 4%, labeled 96%
100[a]	5	500	25	Free 0.24%, labeled 99.86%

Optimization was done to increase the % age of labeled content and decrease the % age of colloidal ^{99m}Tc. a; The Q9P35GC micelles with maximum ^{99m}Tc radiolabeling efficiency were selected for further biodistribution and physical stability studies.

Table S2. Liver to non-target tissue ratios in rabbits (n=5) based on the radiotracer biodistribution calculated as % ID ± S.D.

Time (min)	liver to kidney	liver to lungs	liver to blood
5	2.8 ± 0.4	7.1 ± 0.3	8.6 ± 1.8
10	2.9 ± 0.3	6.2 ± 0.3	12.0 ± 2.0
20	2.4 ± 0.5	17.7 ± 1.3	16.6 ± 2.1
30	3.4 ± 0.3	14.2 ± 0.2	8.2 ± 1.5
60	6.5 ± 1.2	11.6 ± 1.2	7. ± 1.2
120	6.8 ± 1.3	10.5 ± 1.2	6.9 ± 1.3

Table S3. Absorbed dose in rabbit organs over time (0-24 h), following intravenous bolus administration of ^{99m}Tc Q9P35GC micelles (80 ± 2 MBq). Values in rad/MBq, are presented as mean ± SD (n=5).

	Absorbed dose (rad/MBq)	SD
Liver	3.2	0.07
Bladder	2.02	0.04
Spleen	1.1	0.02
Kidney	0.7	0.01
Lungs	0.22	0.004
GIT	0.009	0.0002
